# Hypopituitarism in Traumatic Brain Injury—A Critical Note

**DOI:** 10.3390/jcm4071480

**Published:** 2015-07-14

**Authors:** Marianne Klose, Ulla Feldt-Rasmussen

**Affiliations:** Department of Medical Endocrinology, Copenhagen University Hospital Rigshospitalet, Blegdamsvej 9, DK-2100, Denmark; E-Mail: ulla.feldt-rasmussen@regionh.dk

**Keywords:** head trauma, hypopituitarism, diagnostic criteria

## Abstract

While hypopituitarism after traumatic brain injury (TBI) was previously considered rare, it is now thought to be a major cause of treatable morbidity among TBI survivors. Consequently, recommendations for assessment of pituitary function and replacement in TBI were recently introduced. Given the high incidence of TBI with more than 100 pr. 100,000 inhabitants, TBI would be by far the most common cause of hypopituitarism if the recently reported prevalence rates hold true. The disproportion between this proposed incidence and the occasional cases of post-TBI hypopituitarism in clinical practice justifies reflection as to whether hypopituitarism has been unrecognized in TBI patients or whether diagnostic testing designed for high risk populations such as patients with obvious pituitary pathology has overestimated the true risk and thereby the disease burden of hypopituitarism in TBI. The findings on mainly isolated deficiencies in TBI patients, and particularly isolated growth hormone (GH) deficiency, raise the question of the potential impact of methodological confounding, determined by variable test-retest reproducibility, appropriateness of cut-off values, importance of BMI stratified cut-offs, assay heterogeneity, pre-test probability of hypopituitarism and lack of proper individual laboratory controls as reference population. In this review, current recommendations are discussed in light of recent available evidence.

## 1. Introduction

Traumatic brain injury (TBI) has until recently been considered a rare cause of hypopituitarism, accounting for less than one per cent of all new cases, *i.e.*, less than one new case in 10 million inhabitants per year. Newer studies have indicated that TBI-related chronic anterior pituitary hormone deficiency may be far more frequent, and a recent meta-analysis reported hypopituitarism in over 25% of adults after TBI [1], with similar data found in children. Due to the high overlap of symptoms in TBI and hypopituitarism, posttraumatic hypopituitarism was suggested to contribute substantially to fatigue and other common sequelae after TBI. Most importantly, the need for increased attention in the acute and chronic phases after TBI was underlined as in the case of, e.g., acute adrenal insufficiency, where overlooking the condition can be life-threatening.

Thus, while hypopituitarism after TBI was previously considered rare, it is now suggested to be a major cause of treatable morbidity among TBI survivors. Consequently, in the clinical practice guidelines for the evaluation and treatment of GH deficiency, recommendations for assessment of pituitary function and replacement in TBI were recently introduced [2,3]. Given the high incidence of TBI with more than 100 cases in 100,000 inhabitants, TBI would be by far the most common cause of hypopituitarism if the reported prevalence rates hold true. However, the disproportion between this proposed incidence and the occasional cases of post-TBI hypopituitarism in clinical practice justifies reflection on whether hypopituitarism has been unrecognized in TBI patients or whether diagnostic testing designed for application in high-risk populations such as patients with obvious pituitary pathology or pituitary surgery has overestimated the true risk and thereby the disease burden of hypopituitarism in TBI. The findings in the published studies concerning mainly isolated hormone deficiencies in patients with TBI, in particular isolated growth hormone (GH) deficiency, raises the question of methodological confounding. Thus, the studies cover a broad range of variability of test-retest reproducibility, choice of cut-off values, use or not of BMI stratified cut-offs, assay heterogeneity, pre-test probability of hypopituitarism and lack of proper individual laboratory controls as reference population.

In this review, the published clinical studies on posttraumatic hypopituitarism are scrutinized, and current recommendations are discussed in light of recent available evidence.

## 2. Hypopituitarism in TBI—Pathophysiology

The pathophysiology of anterior pituitary hormone deficiency following TBI remains incompletely understood, but current evidence has indicated a role is played by both direct mechanical injury and injury from hypotension, hypoxia, anaemia and brain swelling causing restriction of flow in the long hypophyseal portal vessels. Support of this concept comes from autopsy studies where up to one-third of patients with a fatal head trauma had anterior pituitary gland necrosis [4,5]. It is unclear whether data from fatal cases can be generalized to explain long-term hypopituitarism in TBI survivors. While pituitary stalk transections will undoubtedly cause hypopituitarism, the extent of pituitary damage needed to induce hypopituitarism is unknown. Imaging techniques appropriately visualizing microstructural damage in the hypothalamic/pituitary region may prove useful, but until then we are fully dependent on clinical presentation and especially biochemical pituitary assessment.

## 3. What is the Reported Prevalence of Acute Anterior Pituitary Hormone Deficiency?

Acute severe illness is a major stress to the body where increased plasma adrenocorticotrophic hormone (ACTH), cortisol, GH and prolactin concentrations, together with low gonadotroph hormones and low triiodothyronine syndrome have all been described as part of important adaptive stress responses in the early phases following trauma, surgery and severe medical illness [6]. Hypoadrenalism during the acute phase may have a major impact for the patients, and it is therefore of special clinical interest. Cohan *et al.* [7] reported that 50% of patients with moderate-to-severe TBI had at least transient adrenal insufficiency in the acute phase (defined by a total cortisol concentration below the 25th percentile of extracranial trauma patients) which was associated with lower blood pressure and higher vasopressor use. They advised that consideration should be given to monitoring of cortisol levels in intubated TBI patients. In a subsequent study, Hannon *et al.* [8] reported a frequent occurrence of acute hypocortisolaemia and central diabetes insipidus in patients with acute TBI, and they could predict mortality. In 13 of 15 TBI patients who developed hyponatraemia during intensive care, cortisol deficiency was suggested by a total plasma cortisol <300 mmol/L, and hyponatraemia in these patients responded to glucocorticoid treatment. Notably, none of the patients had developed warning signs of adrenal insufficiency such as hypotension or hypoglycaemia. In the study by Hamrahian *et al.* [9] nearly 40% of critically ill patients with hypoproteinaemia had subnormal total cortisol despite normal adrenal function as measured by free cortisol concentrations. This was in keeping with findings by Kleindienst *et al.* [10] who described how the adaptive response to critical illness with significantly elevated cortisol levels on admission and subsequent decreased levels in patients ventilated for more than 24 h was attenuated following severe TBI. However, the coincidence of low serum cortisol and increased urinary excretion of glucocorticoid metabolites in about 80% of patients challenged the relevance of using basal total cortisol measurement for evaluation of adequate glucocorticoid production. Profound and variable changes occur during the first week after admission for critical illness in the hypothalamo-pituitary-adrenal (HPA)-axis, including HPA-activation [11], reduction in cortisol degradation [12], as well as lowering of cortisol binding globulin [9,13], and increased tissue sensitivity to glucocorticoids [14]. Furthermore, cortisol is secreted in pulses with diurnal variability which may be abolished in acute critically ill patients [15,16]. All these changes make assessment extremely difficult as common biochemical definitions of hypoadrenalism cannot be used [9,17]. Therefore, the threshold that best describes the patients in need of acute or chronic glucocorticoid replacement is still to be defined and is likely to depend on the underlying illness.

Few longitudinal studies have been designed to evaluate a relationship between acute and long-term pituitary hormone status after TBI. In the acute phase, Agha *et al.* [18] found secondary gonadotropin, GH, ACTH and TSH deficiency in 80%, 18%, 16% and 2%, respectively, of 56 patients with moderate or severe TBI. At one-year follow-up, hormonal abnormalities had recovered in most patients, whereas others had developed *de novo* deficiencies, and although persistent GH and ACTH deficiency was associated with more severe hyposecretion of both GH and cortisol during the acute phase, the authors were unable to identify biochemical predictors of long-term hypopituitarism. Tanriverdi *et al.* [19] reported acute phase pituitary hormone deficiency in 50% of 52 evaluated patients, primarily affecting the gonadothroph axis. However, individual data showed no obvious relationship between early and late pituitary dysfunctions. Klose *et al.* [20] described acute hormone alterations in 76% of 46 patients, and the patients suffering the most severe TBI exhibited the highest prevalence of alterations mimicking hypogonadotropic hypogonadism, central hypothyroidism, hyperprolactinaemia, and increased HPA activity, all in agreement with alterations seen in non-pituitary critical illness [21].

To conclude, pituitary-related hormone concentrations in TBI patients are very commonly affected in the acute setting, but most of them are identical to those seen in non-pituitary critical illness. Most cases recover and it is thus likely that the observed changes predominantly reflect normal physiological changes related to critical illness, for which there is no evidence to support a clinical benefit from hormonal replacement therapy with GH, thyroid hormone, or reproductive hormones. Post-TBI acute hypopituitarism may, however, develop in rare cases and should be considered in the appropriate clinical context. Upon suspicion of hypoadrenalism, an immediate therapeutic trial of glucocorticoid replacement should be commenced, since proper biochemical diagnosis is unreliable in the acute phase after injury. The subsequent treatment response should guide the decision on further treatment and follow-up. In this context, it should be emphasized that patients may present with more subtle signs and symptoms, and still have their lives threatened by undiagnosed hypoadrenalism.

## 4. What is the Reported Prevalence of Chronic Anterior Pituitary Hormone Deficiency?

**Figure 1:**
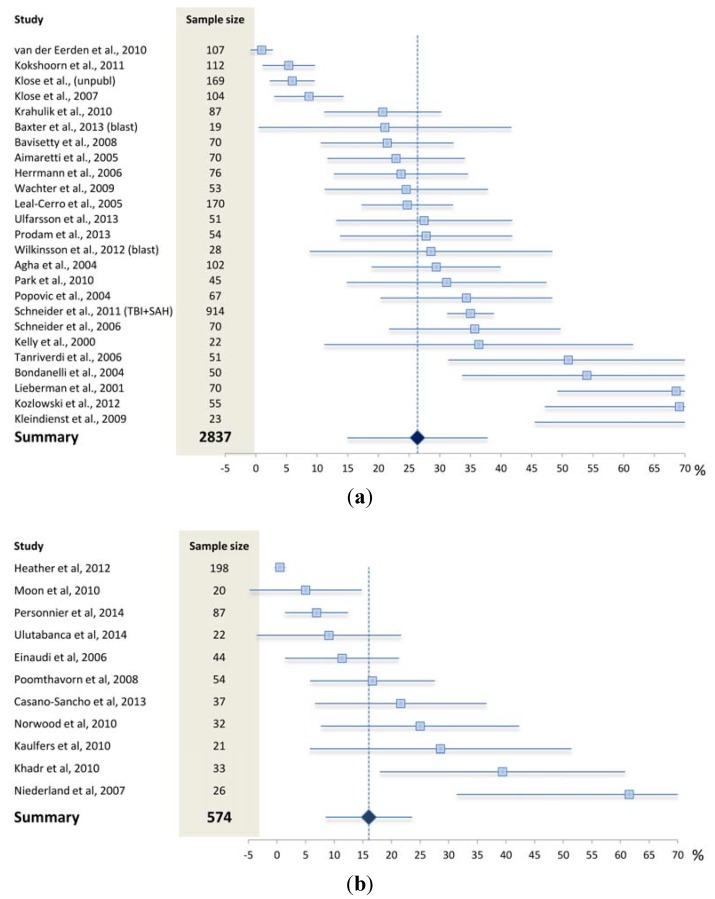
Prevalence rates with 95% confidence intervals in studies assessing pituitary dysfunction after traumatic brain injury in (**a**) adults and (**b**) children/adolescents. As indicated by the width of the confidence intervals, most of the available studies were based on rather small populations. Plot produced according to Neyeloff *et al.* [36].

Several prevalence studies on post-TBI chronic hypopituitarism have been conducted over the last 15 years as summarized in Figure 1. As given in Figure 1A, the overall prevalence from studies performed in adults was 26% which is very similar to results from the meta-analysis by Schneider *et al.* [1]. However, the rate of hypopituitarism varied considerably from negligible to well above 50% in adults, a diversity most probably reflecting methodological differences in terms of patient selection, study designs, and diagnostic procedures, as will be discussed in the following. Very similar data to those in adults were obtained from the paediatric studies (Figure 1B).

### 4.1. What May Affect the Reported Prevalence Rate—Patient Selection?

**Figure 2:**
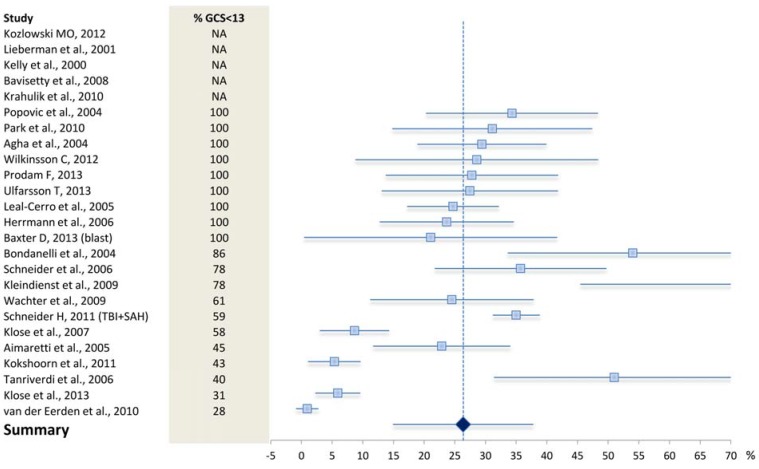
Prevalence rates with 95% confidence intervals in studies assessing pituitary dysfunction after traumatic brain injury in adults. Studies are ranked according to percentage of patients with GCS < 13 (*i.e.*, moderate to severe TBI). Plot produced according to Neyeloff *et al.* [36]. NA: not available.

The study populations varied considerably in terms of trauma severity. In some cases only patients with severe TBI as defined by a Glasgow Coma Scale score (GCS) <8 were included [22,23], whereas other studies investigated more general TBI populations [24,25]. Overall, posttraumatic hypopituitarism tended to be less prevalent in studies also including less severely injured patients [26,27]. Concordantly, indicators of increased trauma severity including low Glasgow Coma Scale, diffuse brain swelling, hypoxia/hypotension, base of skull fractures, increased intracerebral pressure and axonal injury were suggested to be associated with development of hypopituitarism by some [27,28,29,30,31], but not by others [22,32,33]. Ranging the available studies according to percentage of patients with GCS <13, it is clear that trauma severity could not unambiguously explain the observed variation in the rate of hypopituitarism (Figure 2). However, GCS assessing higher cerebral function may not be the best proxy for structural pituitary damage, for which functional MRI might prove more predictive in the future [34]. One study also suggested an aetiological role for presence of antipituitary antibodies [35], which has yet to be confirmed by other investigators.

### 4.2. What May Affect the Reported Prevalence Rate—Diagnostic Challenges?

Pituitary assessment was previously restricted to the classic high risk conditions, with a high *a priori* likelihood for disease, such as patients with obvious pituitary pathology and those undergoing pituitary surgery, who most often suffer from multiple pituitary hormone deficiencies. In recent years, TBI and subarachnoid haemorrhage (SAH) have been added to the list of high risk populations to be considered for pituitary assessment [2,3]. The evidence for their entry into the recommendations came from small studies, with only few of them performing confirmatory testing, where predominantly isolated deficiencies and in particular isolated GH deficiency was reported mainly based on single testing. This has raised suspicion of overt misclassification by applying a diagnostic program tailored for high-risk populations.

**Figure 3:**
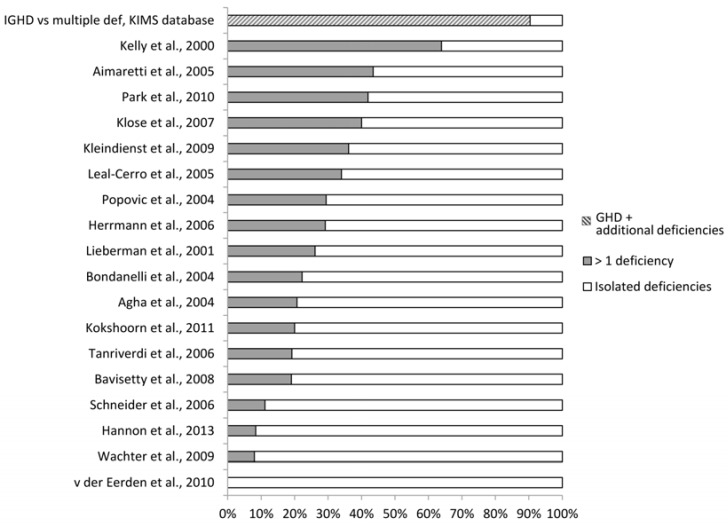
Relative proportion of reported isolated *vs.* multiple (>1 axis) pituitary deficiencies in TBI. The all-cause proportion of isolated GH deficiency (IGHD) in adult hypopituitary patients reported from the KIMS database is included for comparison [52].

Diagnostic uncertainties are plentiful in general, but of special interest in post-TBI hypopituitarism, given that the large majority of these patients were reported with isolated deficiencies and in particular isolated GH deficiency (Figure 3). The diagnosis of hypopituitarism relies on basal and stimulated anterior pituitary and peripheral target hormone concentrations, and diagnostic test-panels and criteria suggesting hypopituitarism have been defined mainly for patients with known hypothalamo-pituitary pathology. Both diagnostic criteria and cut-off points are arbitrary, and grey-zones for each pituitary hormone exist. Thus, test results must be interpreted in the light of pre-test probability and clinical features [37]. Unfortunately, the clinical symptoms in hypopituitarism are most often vague and non-specific, and the diagnostic decision therefore relies on the pre-test probability of disease. The diagnostic process is furthermore highly challenged by vastly different assays for hormone measurements, all with their own reference range, as well as significantly different cut-off levels between normal persons and patients with pituitary deficiency. Establishment of local diagnostic cut-off points are thus required and essential for proper interpretation [24], but most often lacking. In most centres hypopituitarism is a rare condition, and ensuring their own normative data for all the pituitary tests is cumbersome and expensive, which is why many physicians rely on “standard” cut-off limits reported in the literature from other laboratories. Ideally, assessment of TBI patients should be restricted to a few highly specialised collaborative centres using stringent diagnostic criteria for hypopituitarism including, the above mentioned measures as well as obligatory confirmation tests in case of an insufficient test outcome. The latter is highly important due to intraindividual test variation in normal people [38,39], and possible transitory changes due to non-pituitary related stress. For logistic and a variety of other reasons, such recommendations are rarely followed, although the price for the patients may be an incorrect diagnosis and unnecessary treatment.

The diagnosis of GH deficiency is challenged by several confounding factors. Because of GH pulsatile secretion, stimulation tests are needed for the diagnosis to be established, unless the patient has 3 or more additional pituitary deficiencies and a low IGF-I where the probability of having GH deficiency is above 95% [2]. The most common and important confounder for the diagnosis of GH deficiency is obesity, which is a state of relative GH deficiency. In obesity, spontaneous GH secretion is reduced, clearance is enhanced, and stimulated GH secretion is reduced; all of which are reversed upon weight loss [40,41]. The impact of obesity on the diagnostic property differs between the available tests. Obesity may relate to impaired glucose tolerance which influences the ability to achieve adequate hypoglycaemia after insulin administration. Thus, the relative weight-adjusted dose of insulin required to achieve hypoglycaemia during the insulin tolerance test (ITT) is often higher, which is however reflected by the fasting glucose level [42]. Once adequate hypoglycaemia is achieved, the ITT cut-off defining GH deficiency seems unaffected by obesity; however, ITT reproducibility is a problem [43,44] probably relating to pre-test spontaneous GH pulses limiting the remaining secretory capacity [43]. Tests combining GHRH with arginine, pyridostigmin or GH secretagogues are becoming increasingly used in clinical practice, due to the lack of reproducibility, contraindications and precautions related to the ITT. These tests are all very potent but also highly affected by obesity, and more so than the ITT. The need for BMI stratified cut-offs are now recognised to be of the utmost importance, and test specific BMI related cut-offs were recently published [45,46]. Similar limitations were recently published for the glucagon test [47]. In this context, it has to be realized that most of the early studies on posttraumatic hypopituitarism were performed prior to availability and considerations of BMI related cut-offs. Whether or not this might have influenced the reported prevalence remains speculative, but it is noteworthy that the most common and significant predictor of GH deficiency in the early studies was BMI [48,49], supporting the suspicion that obesity may have influenced the test outcome and consequently the reported prevalence rates in a circle conclusion. Also the diagnosis of androgen deficiency may be confounded by several related comorbidities including obesity, diabetes, depression and opioid treatment. Decreased testosterone concentrations in obesity and diabetes mellitus are linked to decreased sex hormone binding globulin (SHBG) [38], whereas opioids may cause gonadal inhibition at the hypothalamic, pituitary and end-organ level [50]. Anticonvulsants used in TBI patients for treatment of pain and epilepsy may, among other things, influence testosterone metabolism via induction of hepatic enzymes in men [51]. However, as GH deficiency, hypogonadism and obesity display a mutual relationship, it may be difficult to discern which dysfunction is causal if any; thus, distinguishing between organic GH and gonadotropin deficiency and obesity is difficult or even impossible, and poses a major diagnostic problem in suspected hypopituitarism in general and in isolated deficiencies in particular.

In addition to confounding, patients labelled as having TBI-related isolated hypopituitarism are prone to being victims of false-positive testing. Given that several axes are assessed in each patient the cumulated risk of false-positive testing is further increased. No diagnostic test for hypopituitarism has 100% sensitivity and specificity. Guidelines have provided cut-points ensuring that cases are not missed which is relevant in high risk populations. The cut-off definition to define deficiency *vs.* normality is however always a trade between sensitivity and specificity. Thus e.g., an ACTH stimulated 30 min. plasma cortisol above 550 nmol/L effectively excludes HPA deficiency, but at the expense of false positive cases where up to 15% not passing 550 nmol/L will have normal adrenal function with a false positive test [53]. The specificity can be increased by lowering the cut-off, at the expense of sensitivity. Similarly, highly sensitive recommended cut-offs for the GHRH-arg test [45] were established to avoid overlooking deficiency, at the expense of specificity, which was only 75% in overweight and obese subjects, again implying that one in four not passing the cut-off will be normal but with a false positive result. Due to the diurnal rhythmicity of testosterone secretion morning, samples are required, but even if morning samples are available in case of a low or low-normal testosterone concentration, sampling must be repeated, because 30% of such men may have a normal testosterone level on repeat measurement [38].

**Figure 4:**
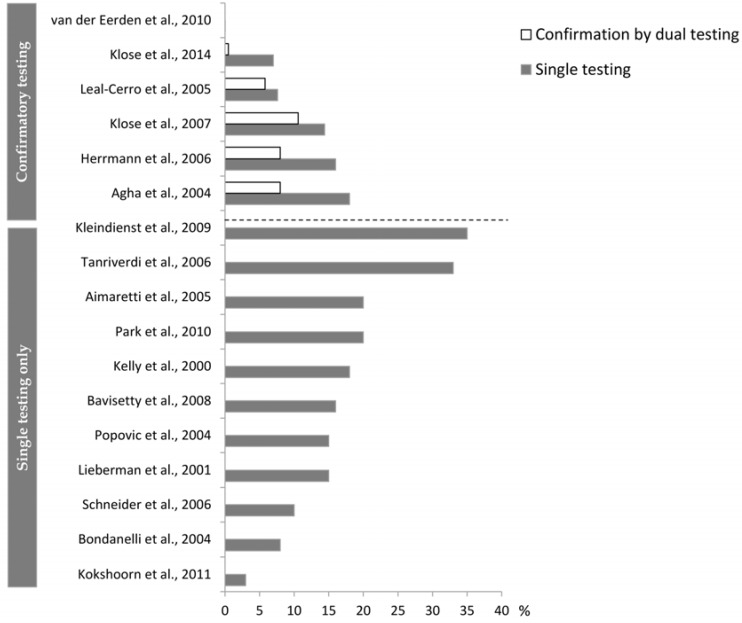
Prevalence of GH deficiency in studies performing single testing *versus* studies adding a subsequent confirmatory test. The prevalence from both the initial test and the subsequent confirmation test are given for studies performing confirmatory testing.

If we accept a relatively low prevalence of posttraumatic hypopituitarism when set against the high prevalence rates for TBI, then a false-positive result will be far more common than a true positive one. In this setting, additional tests to confirm or refute the diagnosis are of paramount importance. However, the requirement of confirmatory testing has been omitted in recent guidelines for the diagnosis of GH deficiency [2]. Whereas this may not be of great importance in high risk populations, we consider it problematic that similar recommendations were transferred to the new indications, including TBI and SAH, where the evidence for considering the patients “high-risk patients” was most often based on single unconfirmed testing, with isolated deficiencies, and using non-BMI stratified cut-offs. The fairness of such concerns was evident in a recent study showing a low concordance of repeated testing for GH deficiency [24], as well as in other studies applying repeated testing in TBI cohorts (Figure 4). Of interest is the fact that, as in TBI, the risk of hypopituitarism after SAH has lately been questioned [54,55,56].

## 5. Implication of Posttraumatic Hypopituitarism

While most TBI patients recover within three months, many are left with significant functional limitations or psychosocial morbidity. Thus, impairments such as depression, anxiety, fatigue, sexual dysfunction, pain, sleep disorders, cognitive dysfunction and decreased health related quality of life (QoL) are all commonly described in TBI survivors. Hypopituitary symptoms highly overlap those observed in TBI patients and concerns have been raised whether undiagnosed, and thus untreated hypopituitarism may contribute to TBI morbidity. The magnitude of such a contribution has still not been defined, and data are conflicting.

Increased disability, decreased QoL and a greater likelihood of depression has been described in patients with posttraumatic GH deficiency [31,57], although others have suggested that neuropsychological and QoL deficits are more closely associated to presence of haemorrhagic lesions on CT than to hypopituitarism *per se* [58]. In a cohort of 104 TBI patients hospitalised at a tertiary hospital with neurosurgical facility, we found that when adjusted for confounders such as trauma severity, posttraumatic hypopituitarism remained an independent predictor of worsened QoL [59], which could point to an association. However, in a very recent population-based study including 463 patients hospitalised at any Danish hospital facility for at least 24 h with evidence of TBI in terms of loss of consciousness, amnesia, or cranial/cerebral imaging abnormalities, thus representing a broader unselected TBI population, we were unable to reproduce such an association between hypopituitarism and QoL [60]. QoL is increasingly used for patient assessment and treatment evaluation. It is a complex entity integrating the patient’s physical, mental and social well-being, and by virtue of its nature, susceptible to many often co-existing conditions. TBI patients often receive medical treatment by antidepressant, opioids and antiepileptic drugs reflecting persistent physical and mental sequelae to the index trauma, which may all be more important for QoL than any hormonal deficiency *per se*. Symptom clusters including fatigue, depression, pain and sleep disorders strongly predict lower QoL in various disease states other than pituitary deficiency [61,62]. This may explain the modest or even absent associations between hormonal deficiencies and QoL after adjustment for above co-variates with possible overpowering dominant influence [60]. Bondanelli *et al.* [63] found peak GH to be an independent predictor of poorer outcome as measured by rehabilitation scales evaluating cognition, disability and functional dependency. Such association between cognitive function impairment and GH axis integrity was later questioned by Pavlovic *et al.* [64] who applied a very extensive neuropsychological battery, selected for high sensitivity for subtle brain dysfunction. No differences were found comparing patients with GH deficiency and those with normal GH function, and no correlation was found between neuropsychological variables and stimulated peak GH or IGF-I levels.

Assessing the physical impact, we described posttraumatic hypopituitarism as an independent predictor of the classical phenotypical features of hypopituitarism, including an unfavourable lipid and body composition profile, and pituitary-insufficient as opposed to sufficient patients showed a higher age, gender, and TBI severity adjusted increase in total cholesterol, BMI and waist circumference [59]. Further, Mossberg *et al.* [65] described reduced aerobic and endurance capacities in patients with posttraumatic isolated GH deficiency as compared to those with normal pituitary function. In children the association between GH deficiency and growth velocity was suggested by some [66], but not by others [67]. In a large population of 198 survivors of structural TBI sustained in early childhood, Heather *et al.* [68] described subnormal peak stimulated GH in 8%, in the context of normal IGF-I and normal growth.

Discrepancies in the presented reported outcome data are likely explained by lack of power, variable diagnostic validity questioning the grouping of patients, and probably most importantly problems correcting for the enormous heterogeneity of co-morbidities in TBI patients. As for QoL, use of different patient-reported outcome questionnaires further complicates direct comparison.

## 6. Treatment of Posttraumatic Hypopituitarism

Kopczak *et al.* [69] screened 509 patients with TBI and SAH for hypopituitarism and found that laboratory values possibly indicating hypopituitarism were common but most patients were clinically not diagnosed as pituitary insufficient and were thus not considered for hormone replacement therapy. There are limited data on a possible treatment effect in patients with anterior pituitary hormone deficiency. Three studies have compared clinical and other outcome variables measured at baseline and after one year of GH replacement in TBI patients compared with outcomes in patients with non-functioning pituitary adenoma (NFPA) from the Pfizer International Metabolic (KIMS) database [70,71,72]. At one-year follow-up, IGF-I SDS levels had increased to the normal range, and improved QoL was observed, in TBI as in NFPA patients, suggesting that TBI patients with GH deficiency benefit from GH replacement in terms of improved QoL in a similar fashion as do NFPA patients. The strength of these KIMS studies was related to the size of the cohorts and the inherent ability to perform direct comparison with NFPA patients, but it is worth mentioning that such registry studies include non-randomised highly selected cohorts which may not be representative for a general cohort of TBI patients. Furthermore, only GH-deficient patients were included and not hypopituitarism as such, and all were treated with GH replacement. In a small study by High *et al.* [73], 23 patients with posttraumatic GH deficiency were randomised to either a year of placebo or GH replacement therapy. Data suggested that some of the cognitive impairments observed in these patients might be partially reversible with appropriate replacement therapy. Similar observations were recorded by Maric *et al.* [74]. Reimunde *et al.* [75] compared the effect of daily neurocognitive rehabilitation plus GH replacement to neurocognitive rehabilitation alone and reported larger scale improvements in the GH-deficient TBI patients on a combined treatment schedule. Although this is encouraging, there is still inadequate evidence to demonstrate that pituitary replacement therapy improves the metabolic profile, neuro-cognitive symptoms, psychosocial problems, or work-related activities in TBI patients, and larger randomised placebo-controlled studies are much awaited and crucial for proper evidence-based clinical decisions.

## 7. Conclusions

Anterior pituitary hormone alterations are frequently encountered in the acute phase after TBI. The relevance and therapeutic implications of such endocrine changes are still debated. Routine acute phase assessment of the growth hormone, thyroid, and gonadal axes cannot be recommended, as there is currently no evidence of a clinical benefit of replacement therapy at this stage resembling general critical illness. However, since untreated adrenal insufficiency can be life threatening, and as correct biochemical assessment is difficult in the acute phase, the diagnosis should be based on the clinical picture, and immediate treatment instituted upon suspicion.

Although current guidelines have included TBI as an indication for hypopituitary testing in the chronic phase >1 year after TBI, recent data cast doubt on the evidence for such recommendations of general pituitary assessment, and indicate a need for re-evaluation of guidelines in order to diminish the financial burden of routine pituitary testing in patients with TBI. Given the high incidence of TBI, this has an important general interest as reliable predictors and screening tools still remain to be identified. In patients chosen for pituitary assessment, we advise for stringent testing, and given the complexity of the diagnosis where several confounding factors have to be taken into account, assessment should ideally be restricted to few highly specialised collaborative centres using high-quality diagnostic criteria for hypopituitarism as well as obligatory confirmation tests in case of an insufficient test outcome. Confirmatory testing of all pituitary axes has often been neglected, but is highly recommended to avoid overdiagnosing, which is of particular importance in TBI and other patient groups with low *a priori* likelihood of pituitary hormone deficiency. Causality is still to be shown, and whether hormonal substitution therapy could be of additional relevance in cases of mild deficiencies needs to be proven. However, in cases of overt posttraumatic multiple pituitary hormone deficiencies, a treatment effect of similar efficacy as in any other cause of pituitary deficiency is expected and thus should be instituted based on normal clinical and biochemical criteria.
